# More about the Cowdray Gifts

**Published:** 1921-01-29

**Authors:** 


### More about the Cowdray Gifts.

The more that becomes known of the great gifts to the College of Nursing which Viscount and Viscountess Cowdray have made, the more is it possible to appraise their exceeding value to the nursing profession as a whole, and indirectly to the health of the nation, which is bound to be influenced beneficially by the improved status, increased educational opportunities, and greater social freedom which these gifts confer. The house, 20 Cavendish Square, is to be used exclusively as a Club, to be known by the wish of the Council as the Cowdray Club. The garden adjoining, having frontage to Henrietta Street, is to be utilised as a site for the College. The conversion of the Club is to be begun immediately, and it is hoped to have it completed by Midsummer, but it is proposed to defer the erection of the College until building conditions become easier. Meanwhile, Lady Cowdray has advised the Council that the gift will be increased to a total of £100,000, and that interest on that sum will be accumulating for the College. The College will contain offices, class-rooms, laboratories, lecture halls, etc., while the upper floors will provide over fifty bedrooms, having direct access to the Club. It is a noble gift, worthy of the great ideal which Lord and Lady Cowdray have for the nursing profession, to which they have given practical expression already in numberless ways.

We are glad to learn that the scheme of the College Club does not contemplate restricting the membership within limits which might militate against the Club's success, and minimise its value to the profession. Hence, though preferential treatment is secured to members of the College of Nursing, the College Club will be open to all nurses and to women members of other professions. This is all to the good. Nurses have led far too narrow a life and their interests have necessarily been too much confined to the atmosphere, surroundings and outlook of the sick and those who treat them. Intercourse with one's fellows broadens the individual outlook and adds to the savour of life. It is not surprising, therefore, that the London Centre meeting on January 25 gave an enthusiastic approval to the wise provision of the Council that the Club shall be of international character, open by election to women of other professions than that of nursing.

